# Sex Differences in Clinical Parameters, Pharmacological and Health-Resource Utilization in a Population With Hypertension Without a Diagnosis of COVID-19

**DOI:** 10.3389/ijph.2022.1604913

**Published:** 2022-08-25

**Authors:** Ana Lear-Claveras, Bárbara Oliván-Blázquez, Ana Clavería, Sabela Couso-Viana, Jesús Puente-Comesaña, Rosa Magallón Botaya

**Affiliations:** ^1^ Aragonese Research Group in Primary Care (Grupo Aragonés de Investigación en Atención Primaria/GAIAP), Aragón Health Research Institute, Zaragoza, Spain; ^2^ Department of Psychology and Sociology, Faculty of Social Sciences, University of Zaragoza, Zaragoza, Spain; ^3^ Network for Research on Chronicity, Primary Care, and Health Promotion (RICAPPS), Barcelona, Spain; ^4^ I-Saúde Group, Galicia South Health Research Institute, Vigo, Spain; ^5^ Vigo Health Area, SERGAS, Vigo, Spain; ^6^ Department of Medicine, Psychiatry and Dermatology, University of Zaragoza, Zaragoza, Spain

**Keywords:** utilization, lockdown, pharmacoepidemiology, lifestyle behaviours, hypertension (HTN), COVID-19 epidemic

## Abstract

**Objectives:** Determine the changes in clinical, pharmacological and healthcare resource use parameters, between the 6 months prior to the lockdown and the 6 months following its end, in a population with hypertension who did not have a diagnosis of COVID-19.

**Methods:** Real world data observational study of 245,979 persons aged >16 years with hypertension in Aragon (Spain). Clinical (systolic-diastolic blood pressure, estimated glomerular filtration rate (eGFR), blood creatinine, cholesterol, triglycerides and anthropometric measures); pharmacological (diuretics, calcium channel antagonists, and ACE inhibitors); and utilization of healthcare resources were considered. We performed the Student’s T-test for matched samples (quantitative) and the Chi-squared test (qualitative) to analyze differences between periods.

**Results:** SBP, DBP, parameters of renal function and triglycerides displayed a significant, albeit clinically irrelevant, worsening in women. In men only DBP and eGFR showed a worsening, although to a lesser extent than in women. Certain antihypertensive drugs and health-resource utilization remained below pre-pandemic levels across the 6 months post-lockdown.

**Conclusion:** Changes in lifestyles, along with difficulties in access to routine care has not substantially compromised the health and quality of life of patients with hypertension.

## Introduction

Cardiovascular diseases are the leading cause of morbidity and mortality globally, and in 2019 accounted for 17.9 million deaths, almost one third of the sum total [1]. Among these diseases, complications of arterial hypertension (AHT) (defined as systolic blood pressure (SBP) ≥140 mmHg and diastolic blood pressure (DBP) ≥90 mmHg measured in a doctor’s office) cause 9.4 million deaths each year [2, 3].

AHT is one of the most prevalent chronic diseases, affecting over one billion persons worldwide [4]. In Spain, some 14 million adults have high blood pressure (BP) (33%), with prevalence of this disease rising to double (66%) among persons over 60 years of age [3]. According to the Aragon Regional Health Authority, in 2018, 19% of men and 20% of women had a diagnosis of AHT, percentages that rose to 60% among those over the age of 65 [5]. With over 1,300,000 inhabitants, Aragon has a rapidly aging population, with this phenomenon being more pronounced in rural than in urban areas. The capital, Zaragoza, concentrates over 80% of the population, while rural towns with fewer than 2,000 inhabitants, which represent 92% of the region’s urban centers, account for only 16% of the population [6].

A number of chronic conditions, including AHT [7], have been associated with a worse COVID-19 disease course. Even so, this relationship would not seem to be altogether clear for this disease, i.e., although there are several studies which suggest that persons with high blood pressure present with a higher risk of poor prognosis and mortality in the case of COVID-19 [8, 9], this association could well be confounded by age [7, 10]. AHT is very frequent among advanced age groups, which have undeniably proven to be especially vulnerable to the new virus SARS-CoV-2. There is, however, sufficient evidence to show a higher risk of hospital mortality due to COVID-19 among patients with underlying complications stemming from high blood pressure levels (such as heart failure, kidney disease or stroke) [11–13].

Good management and control of blood pressure during the pandemic is fundamental to reduce both the burden of this disease, and any ensuing cardiovascular complications and worse prognosis among infected patients [7]. Prior to the COVID-19 outbreak, health professionals at primary care (PC) centers played an important role in the promotion of healthy lifestyles [14], by trying to ensure the prevention, early detection and control of this and other chronic diseases [15]. Once BP levels have been successfully stabilized, the standard follow-up recommended for such patients is a check by nursing staff every 3–6 months, and an annual examination by the physician or a joint examination by both professionals [16]. The outbreak of the pandemic in March 2020 modified the functions of PC teams, a development that possibly had an impact on the follow-up of patients with AHT. Since healthcare services were forced to focus their resources on diagnosis and treatment of COVID-19 [17], this entailed a reduction in routine face-to-face care of persons with AHT and other chronic diseases [18].

Furthermore, AHT is strongly influenced by virus control measures [19]. On 15 March 2020, the Spanish government declared a nationwide state of alarm, thereby placing the public under home confinement until 3 May. By limiting the possibility of engaging in physical activity, these measures could thus have increased sedentary behaviors among these patients [20]. Similarly, isolation could generate feelings of loneliness and increase levels of anxiety or stress [21], negative feelings which are associated with developing unhealthy lifestyles, such as the intake of hypercaloric diets [22], or tobacco and alcohol consumption [23, 24].

The implications of COVID-19 therefore go beyond the harm related with the infection itself. Less rigorous control of blood pressure, coupled with the adoption of unhealthy lifestyles during strict lockdown, could increase the likelihood of suffering some complication or cardiovascular event, something that would translate as an increase in the burden of these diseases in the medium term.

The great majority of studies on AHT and COVID-19 have investigated the influence exerted by this disease on the prognosis of infected patients [7–10], as well as the association between antihypertensive drugs and risk of infection [25]. Far fewer studies have analyzed the impact of the pandemic on the uninfected population with AHT [26–28]. Hence, the aim of this study was to analyze changes in clinical parameters, use of antihypertensive drugs, and health-resource utilization between the 6 months pre-lockdown and the 6 months post-lockdown, among patients older than 16 years in Aragon who were shown to be diagnosed with AHT in their electronic medical records (EMR) but who did not have a diagnosis of COVID-19 during the study period.

## Methods

### Study and Design Population

A real world data observational study of the population over the age of 16 years in a region in the north of Spain, Aragon (*n* = 1,122,151). To ascertain the repercussions of lockdown measures on the health of the population with hypertension (*n* = 259,808), we decided to include only those patients >16 whose EMR showed diagnosis of AHT as per the International Classification of Diseases 10th Revision [29], who during the months of study, did not have a diagnosis of COVID-19 (*n* = 245,979).

For each individual, we obtained EMR data across the 6 months immediately before lockdown (14 September 2019–15 March 2020) and the 6 months immediately after lockdown (03 May 2020–04 November 2020).

### Data-Sources

This study was based on data sourced from longitudinal EMR of primary care in Aragon.

EMR are widely established in Spain; and in the case of Aragon, implementation of EMR in the region’s health system concluded in 2011. All data generated in the healthcare process (PC and hospital) of patients enrolled in Spain’s national health system, which has a population coverage of over 90%, are pooled and shared by all the professionals in the system.

### Variables

The following sociodemographic variables were included in this study: sex, age, pharmaceutical services and rurality of the health zones (rural or urban with less or more than 10,000 inhabitants). In addition, we also considered the number of deaths in the study population for each of the periods measured.

For study purposes, we recorded the number of comorbidities and chronic comorbid conditions with prevalences higher than 5% [30] 1) somatic comorbidities: arrhythmias, heart failure, ischemic heart disease, dyslipidemias, obesity, overweight, vascular diseases, cerebrovascular disease, diabetes, chronic bronchitis, chronic obstructive pulmonary disease (COPD), asthma, chronic kidney disease, hypo- and hyperthyroidism, anemia, neoplasm, hearing loss, cataracts, glaucoma, osteoarthritis, osteoporosis, dorsopathy and 2) psychological comorbidities: smoking habit, alcoholism, insomnia, anxiety and depression, autolytic attempt, and dementia.

We selected clinical and analytical parameters related with AHT (SBP, DBP, estimated glomerular filtration rate (eGFR), blood creatinine, total cholesterol, low-density lipoproteins (LDL), high-density lipoproteins (HDL), triglycerides, waist circumference, body weight, and body mass index (BMI)).

Variations in drug treatment were evaluated by reference to changes in the daily human doses (DHD) for each period, as shown by retail pharmacy dispensing. DHD are calculated on the basis of the standard daily defined dose (DDD) [a measure stipulated by the World Health Organization (WHO)] and other parameters, in accordance with the following formula:

DHD=Registered consumption of the active ingredient∗1000 inhabitantsStandard DDD*n°inhabitants/period*365 days (1)

Taking into account the Anatomical Therapeutic Chemical (ATC) Classification System, we analyzed the following codes of the drugs of choice for treatment of this disease, as indicated by the Spanish Society for Family and Community Medicine (Sociedad Española de Medicina de Familia y Comunitaria – semFYC –) [31]: CO3 (diuretics); C08 (calcium channel antagonists); and C09A (angiotensin-converting enzyme inhibitors – ACE inhibitors –).

Lastly, health-resource utilization by these patients was evaluated using variables linked to use of PC services (number of patients, and number of nursing and general practitioner (GP) visits for routine or continued care, at the health center or home; and number of patients and number of visits to other health center professionals, such as social workers, physiotherapists or midwives) and specialized services (number of visits to specialized care, number of diagnostic tests performed, number of visits to emergency services, hospitalizations and admissions to intensive care units (ICU), and duration of such stays), for each of the periods covered.

### Statistical Analysis

Owing to the large sample size, parametric tests were used for analysis purposes [32]. We performed a descriptive analysis of the study variables, using frequencies, means and standard deviation.

For the clinical variables we calculated the mean and standard deviation (SD) of the parameters for each of the study time points (pre-lockdown and post-lockdown) by sex. If there was more than one measurement of the same parameter for the same individual, the median and interquartile range (IQR) were calculated. To compare differences in means between the baseline measurement and the measurement at 6 months post-lockdown, we used the Student’s T-test for matched samples.

To determine variations in clinical parameters, the proportion of people who had maintained, worsened or improved their BP values were calculated. People who had remained stable were defined as those who: 1) before and after lockdown presented values in range (I-I) or 2) before and after lockdown presented values out of range (O-O). Those who presented values in range before lockdown and after it presented values out of range were considered as worsening (I-O). Lastly, we considered as improvements those who had values out of range before lockdown and values in range after lockdown (O-I). Values greater than 140 and 90 mmHg for systolic and diastolic blood pressure, respectively, were considered out of range.

We use Chi-squared test for calculate the proportion of people who had maintained, worsened or improved by sex.

To ascertain variations in drug use, we calculated the DHD of the study population for each period, and transformed it into its annual equivalent.

To compares differences in the utilization of healthcare resources, we used the Student’s T-test for matched samples in the case of quantitative variables, and the Chi-squared test in the case of qualitative variables. For those variables with less than 100 observations, a Wilcoxon rank test was used.

## Results

Six months after the end of strict lockdown in Aragon, a total of 245,979 persons over the age of 16 years with diagnosis of AHT in their EMR did not have a diagnosis of COVID-19 during the study period.

The descriptive analysis showed that: 52.1% of the population were women, mean age 70 years (SD: 13.6); over two thirds (68.1%) had an income of under 18,000 euros per year, and a little over half (51%) lived in urban areas. 95.2% of patients with AHT presented with some associated comorbidity, with the category of three related comorbidities being the one that embraced the highest number of individuals (17.9%). Among the chronic comorbidities, the most prevalent were dyslipidemias (53.6%), dorsopathies (30.6%), anxiety and depression (29.2%), neoplasms (28%), and diabetes (23.3%) (Table 1).

**TABLE 1:** Sociodemographic data and chronic comorbidities in patients with hypertension, without diagnosis of COVID-19 (Aragon, Spain. November 2020).

	N (%)
Age	
Mean (SD)	70.0 (13.6)
Sex	
Men	117,924 (47.9)
Women	128,055 (52.1)
Pharmaceutical services	
<18,000	167,611 (68.1)
18,000–100,000	67,958 (27.6)
>100,000	1,147 (0.5)
Free drug prescriptions	6,835 (2.8)
Mutual insurance	2,294 (0.9)
Uninsured	134 (0.1)
Rurality of the health zones	
Urban	125,327 (51.0)
Rural	120,651 (49.0)
Number of comorbidities	
One	27,978 (11.4)
Two	40,629 (16.5)
Three	43,971 (17.9)
Four	40,529 (16.5)
Five	31,465 (12.8)
Six	21,465 (8.7)
Seven	13,556 (5.5)
More than seven	14,475 (5.9)
Chronic comorbidities (Yes %)	
Arrhythmias	25,122 (10.2)
Heart failure	10,661 (4.3)
Ischemic Heart disease	18,659 (7.6)
Dyslipidemia	131,946 (53.6)
Obesity	47,859 (19.5)
Overweight	4,568 (1.9)
Vascular diseases	9,794 (4.0)
Cerebrovascular disease	19,421 (7.9)
Diabetes	57,295 (23.3)
Chronic bronchitis	3,749 (1.5)
COPD^a^	13,590 (5.5)
Asthma	15,104 (6.1)
Chronic kidney disease	28,762 (11.7)
Hypothyroidism	27,333 (11.1)
Hyperthyroidism	12,313 (5.0)
Anemia	38,482 (15.6)
Neoplasm	68,870 (28.0)
Hearing loss	24,660 (10.0)
Cataracts	40,586 (16.5)
Glaucoma	24,938 (10.1)
Osteoarthritis	31,807 (12.9)
Osteoporosis	31,575 (12.8)
Dorsopathy	75,172 (30.6)
Smoking habit	30,368 (12.3)
Alcoholism	3,848 (1.6)
Insomnia	38,183 (15.5)
Anxiety and depression	71,792 (29.2)
Autolytic attempt	458 (0.2)
Dementia	9,853 (4.0)

a Chronic obstructive pulmonary disease.

Of the 259,808 patients older than 16 years with a diagnosis of hypertension 13,829 were not included in the study. 4,485 because they had a diagnosis of COVID-19 during the study period and 9,344 because they died. Of these 4,352 died in the 6 months preceding the declaration of the state of emergency (14 September 2019 through 15 March 2020), and a further 4,992 in the 6 months following the end of lockdown (03 May 2020 through 04 November 2020). Taking into account the Aragon population in the middle of the period in each of the two measurement moments, the mortality rate per 1000 individuals was 3.3 (95%CI 3.2–3.4) and 3.7 (95%CI 3.6–3.9), respectively.

Changes in clinical parameters observed on comparing the baseline measurement with the measurement in the 6 months post-lockdown by sex, can be seen in Table 2. In women the parameters of SBP [*p* < 0.001 (95%CI: −0.69 to −0.37)], DBP [*p* < 0.001 (95%CI: −0.78 to −0.59)], eGFR [*p* < 0.001 (95%CI: 1.62–2.35)] blood creatinine [*p* < 0.001 (95%CI: −0.04 to −0.03)] and triglycerides [*p* 0.016 (95%CI: −4.64 to −0.48)] showed a slight, though significant, worsening with respect to the baseline measurement. In men only DBP [*p* 0.010 (95%CI: −0.24 to −0.03)] and eGFR [*p* < 0.001 (95%CI 0.83–1.64)] showed a worsening, although to a lesser extent than in women. For the rest of variables (total cholesterol, LDL, body weight and BMI) both sexes appeared to undergo a slight but equally significant (*p* < 0.001) improvement. The same trend was observed in HDL only in women [*p* < 0.001 (95%CI: 0.52 – 1.11)] and in triglycerides in men [*p* 0.001 (95%CI 2.19 – 7.95)].

**TABLE 2:** Clinical parameters 6 months pre-lockdown and 6 months post-lockdown (Aragon, Spain. September 2019–November 2020).

	Women					Men				
	N	6 months before	6 months after	95% CI	*p*	N	6 months before	6 months after	95% CI	*p*
		Mean (SD)	Mean (SD)				Mean (SD)	Mean (SD)		
SBP^a^	33,769	135.7 (13.9)	136.3 (15.4)	(−0.69; −0.37)	<0.001	26,126	136.2 (14.0)	136.0 (15.2)	(0.05; 0.40)	0.010
DBP^b^	33,763	75.6 (8.7)	76.3 (9.4)	(−0.78; −0.59)	<0.001	26,124	76.6 (9.3)	76.8 (9.7)	(−0.24; −0.03)	0.010
eGFR^c^	2429	72.8 (21.4)	70.8 (21.9)	(1.62; 2.35)	<0.001	1951	73.3 (20.8)	72.0 (21.5)	(0.83; 1.64)	<0.001
Blood creatinine	2452	0.8 (0.3)	0.9 (0.3)	(−0.04; −0.03)	<0.001	1984	1.1 (0.36)	1.1 (0.4)	(−0.04; −0.02)	<0.001
Total cholesterol	2446	199.0 (41.4)	195.1 (40.3)	(2.48; 5.26)	<0.001	1944	179.7 (40.2)	174.9 (39.5)	(3.26; 6.23)	<0.001
LDL^d^	2252	115.9 (36.3)	112.3 (35.1)	(2.38; 4.84)	<0.001	1761	103.6 (35.5)	100.1 (34.5)	(2.17; 4.84)	<0.001
HDL^e^	2322	56.8 (13.2)	56.0 (12.9)	(0.52; 1.11)	<0.001	1872	48.6 (16.2)	48.2 (12.3)	(−0.19; 0.99)	0.180
Triglycerides	2391	134.3 (68.3)	136.9 (71.2)	(−4.64; −0.48)	0.016	1921	138.3 (83.6)	133.2 (78.0)	(2.19; 7.95)	0.001
Waist circumference	931	100.9 (12.3)	100.9 (12.3)	(−0.29; 0.24)	0.853	857	106.4 (13.0)	106.3 (13.0)	(−0.16; 0.29)	0.563
Body weight	18,976	71.2 (13.2)	70.9 (13.5)	(0.23; 0.33)	<0.001	15,720	83.5 (13.9)	82.7 (14.1)	(0.69; 0.82)	<0.001
BMI^f^	11,689	29.8 (5.2)	29.7 (5.3)	(0.06; 0.12)	<0.001	9824	29.8 (4.4)	29.6 (4.5)	(0.19; 0.26)	<0.001

a Systolic blood pressure.

b Diastolic blood pressure.

c Estimated glomerular filtration rate.

d Low-density lipoproteins.

e High-density lipoproteins.

f Body mass index.

Number and percentage of women and men with a stable evolution, deterioration or improvement in clinical parameters at 6 months is shown in Table 3. For SBP approximately 2/3 remain unchanged (I-I or O-O), with no differences according to sex. For individuals who improve (O-I) there are significant differences between the clinical evolution of the parameters and sex, therefore improvements in SBP and DBP depends on sex. The same differences can be seen in SBP for individuals who worsened (I-O).

**TABLE 3:** Number and percentage of patients with hypertension with a maintain get better or get worse evolution at 6 months (Aragon, Spain. September 2019 – November 2020).

	Remain unchanged: (I-I^a^ or O-O^b^)			(I-O)^c^			(O-I)^d^		
	Women	Men	*p*	Women	Men	*p*	Women	Men	*p*
	N (%)	N (%)		N (%)	N (%)		N (%)	N (%)	
SBP^e^	31856 (73.5)	25014 (73.6)	0.573	6294 (14.5)	4604 (13.6)	<0.001	5219 (12.0)	4353 (12.8)	0.001
DBP^f^	40808 (93.7)	31538 (92.6)	<0.001	1708 (3.9)	1396 (4.1)	0.216	1014 (2.3)	1122 (3.3)	<0.001

a In range before and after lockdown.

b Out of range before and after lockdown.

c In range before lockdown and out of range after it.

d Out of range before lockdown and in range after it.

e Systolic blood pressure.

f Diastolic blood pressure.

With respect to drug use (Table 4), in the 6 months post-lockdown the total number of DHD of diuretics dispensed at pharmacies showed a slight increase in comparison with the previous 6 months, with the exception of two thiazide diuretics (Chlorthalidone and Xipamide) which registered a decrease in this same period. In the case of calcium channel antagonists, the number of DHD of all Dihydropyridines (except Nicardipine and Lercardipine) and Bencilalquilaminas/Phenylalkylamines (Verapamil) fell, in contrast to Benzothiazepines (Diltiazem) which saw a rise in the last 6 months. As regards ACE inhibitors, the total number of DHD also decreased in all the drugs in this group (except Quinapril, Fosinopril and Trandolapril).

**TABLE 4:** Number of daily human doses 6 months pre-lockdown and 6 months post-lockdown. (Aragon, Spain. September 2019–November 2020).

	6 months before	6 months after
	DHD^a^	
Diuretics		
Chlorthalidone	10.45	10.12
Xipamide	0.60	0.58
Indapamide	9.11	9.27
Furosemide	71.86	74.65
Bumetanide	0.11	0.11
Torasemide	13.05	13.45
Calcium channel antagonist		
Amlodipine	59.06	58.78
Felodipine	0.33	0.33
Nicardipine	0.10	2.16
Nifedipine	4.44	3.62
Nimodipine	0.43	0.41
Nisoldipine	0.04	0.03
Nitrendipine	0.35	0.26
Lacidipine	0.51	0.50
Manidipine	20.0	19.56
Barnidipine	5.62	4.91
Lercanidipine	10.77	10.78
Verapamile	1.69	1.67
Diltiazem	10.03	10.55
ACE^b^ inhibitors		
Captopril	2.15	2.09
Enalapril	91.92	88.82
Lisinopril	13.35	13.0
Perindopril	1.96	1.64
Ramipril	63.20	61.10
Quinapril	0.04	1.69
Benazepril	0.06	0.05
Cilazapril	0.06	0.06
Fosinopril	0.02	0.56
Trandolapril	0.17	0.21
Imidapril	5.03	4.64

a Daily human doses.

b Angiotensin—converting enzyme inhibitors.

In terms of health-resource utilization, as Table 5 shows, the total number of patients with AHT but without a diagnosis of COVID-19 who visited PC services in the 6 months after the end of strict lockdown, fell as compared to the 6 months before the beginning of lockdown (*p* < 0.001). Only the number of patients who received home nursing visits (routine care) rose in comparison with the previous 6 months (*p* < 0.001).

**TABLE 5:** Number of patients who used Primary Care services 6 months pre-lockdown and 6 months post-lockdown (Aragon, Spain. September 2019–November 2020).

	6 months before		6 months after		*p*
	N	%	N	%	
No. of nursing patients (routine care) at health center or by telephone	166,214	67.57	152,034	61.81	<0.001
No. of nursing patients (routine care) at home	23,102	9.39	24,871	10.11	<0.001
No. of nursing patients (continued care) at health center	26,115	10.62	20,022	8.14	<0.001
No. of nursing patients (continued care) at home	7,970	3.24	6,225	2.53	<0.001
No. of GP^a^ patients (routine care) at health center or by telephone	215,925	87.78	203,282	82.64	<0.001
No. of GP^a^ patients (routine care) at home	22,656	9.21	17,034	6.92	<0.001
No. of GP^a^ patients (continued care) at health center	43,085	17.51	36,746	14.94	<0.001
No. of GP^a^ patients (continued care) at home	9,529	3.87	6,761	2.75	<0.001

a General Practitioner.

Table 6 shows a decrease in the number of visits to nursing and family medicine services, both in routine and continued care, across the 6 months following the end of lockdown (*p* < 0.05), a decrease that was only significant in terms of the number of home nursing visits made to provide continued care (*p* 0.655, 95%CI: −0.17–0.27). With respect to the number of visits to other health center professionals, these fell solely in the case of physiotherapists (*p* < 0.001, 95%CI: 0.99–1.93), and rose in the case of midwives (*p* 0.003, 95%CI: −0.67 to −0.14) and social workers (*p* 0.002, 95%CI: −0.58 to −0.14).

**TABLE 6:** Number of visits and diagnostic test prescribed 6 months pre-lockdown and 6 months post-lockdown (Aragon, Spain. September 2019–November 2020).

	N	6 months before	6 months after	95%CI	*p*
		Mean (SD)			
No. of nursing visits (routine care) at health center or by telephone	125,990	4.51 (4.91)	3.80 (4.49)	0.69; 0.74	<0.001
No. of nursing visits (routine care) at home	12,472	6.64 (9.71)	6.22 (9.43)	0.26; 0.60	<0.001
No. of nursing visits (continued care) at health center	5,180	2.17 (3.17)	1.91 (2.82)	0.17; 0.34	<0.001
No. of nursing visits (continued care) at home	1,494	2.25 (3.31)	2.21 (3.98)	−0.17; 0.27	0.655
No. of GP^a^ patients (routine care) at health center or by telephone	185,259	5.09 (4.16)	5.03 (4.61)	0.05; 0.09	<0.001
No. of GP^a^ patients (routine care) at home	7,181	3.42 (3.63)	2.68 (2.88)	0.66; 0.82	<0.001
No. of GP^a^ patients (continued care) at health center	11,668	1.79 (1.70)	1.83 (1.95)	−0.07; 0.00	0.079
No. of GP^a^ patients (continued care) at home	1,640	1.73 (1.38)	1.63 (1.26)	0.02; 0.18	0.009
No. of visits to other professionals					
Physiotherapist	905	5.84 (6.16)	4.38 (4.96)	0.99; 1.93	<0.001
Midwife	400	1.98 (1.82)	2.39 (2.17)	−0.67; −0.14	0.003
Dental stomatologist	537	2.15 (1.72)	2.33 (1.77)	−0.38; 0.02	0.084
Social worker	855	2.53 (2.54)	2.88 (3.22)	−0.58; −0.14	0.002
No. of specialized care visits (first visit)	7,430	1.45 (0.84)	1.51 (0.88)	−0.08; −0.02	<0.001
No. of specialized care visits (successive visits)	61,678	2.61 (2.18)	2.53 (2.32)	0.06; 0.10	<0.001
No. of diagnostic test performed					
X-rays	34,541	1.13 (1.25)	0.96 (1.14)	0.15; 0.18	<0.001
Ultrasound	34,541	0.31 (0.55)	0.29 (0.53)	0.01; 0.02	<0.001
Resonance	34,541	0.12 (0.36)	0.13 (0.36)	−0.01; −0.00	0.002
CAT scans	34,541	0.31 (0.62)	0.32 (0.63)	−0.02; −0.01	<0.001
Blood count	43,387	0.31 (0.54)	0.23 (0.49)	0.07; 0.08	<0.001
Biochemistry	43,387	1.01 (0.66)	0.93 (0.67)	0.07; 0.09	<0.001
Microbiology	43,387	0.22 (0.62)	0.28 (0.69)	−0.07; −0.06	<0.001
Immunology	43,387	0.14 (0.39)	0.12 (0.36)	0.02; 0.02	<0.001
Coagulation test	43,387	0.03 (0.19)	0.04 (0.22)	−0.01; −0.01	<0.001
Urine test	43,387	0.35 (0.60)	0.31 (0.58)	0.03; 0.05	<0.001
No. of visits to emergency service	10,302	1.69 (1.24)	1.59 (1.15)	0.08; 0.13	<0.001
No. of hospital admissions	15,199	1.27 (0.67)	0.26 (0.67)	0.99; 1.03	<0.001
No. of days of hospital stay	2,725	15.35 (66.33)	14.91 (65.19)	−0.31; 1.20	0.249
No. of ICU^b^ admissions	5	1 (0.00)	1 (0.00)		^c^
No. of days of ICU^b^ stay	5	101.40 (87.67)	100.20 (89.33)	−2.13; 4.53	0.317^d^

a General Practitioner.

b Intensive Care Unit.

c The correlation ant t cannot be calculated because the standard error of the difference is 0.

d Wilcoxon rank test.

When it came to the number of visits to specialized care, opposite but statistically significant trends were in evidence. Among patients with AHT, the number of first visits to specialized care rose in the 6 months after the end of lockdown (*p* < 0.001, 95%CI: −0.08 to −0.02), while the number of control visits to these same services fell across the same period (*p* < 0.001, 95%CI: 0.06–0.10).

In comparison with the previous 6 months, the number of X-rays, ultrasound, blood count, biochemistry, immunology and urine tests decreased among patients with AHT. In contrast, the number of resonances, CAT scans, microbiology and coagulation tests increased among these patients in the 6 months after the end of lockdown, with all these variations being statistically significant (*p* < 0.05).

Lastly, the number of visits to emergency services (*p* < 0.001, 95%CI: 0.08–0.13) and the number of hospitalizations (*p* < 0.001, 95%CI: 0.99–1.03) also decreased, with no statistically significant differences being found between the number of ICU admissions and the duration of hospital stay.

## Discussion

The results of our study suggest a clinically irrelevant slight worsening in blood pressure levels in the 6 months after the end of the lockdown, especially in women. These results are similar to the results of other articles [26, 27] that also show small variations or even improvement in blood pressure levels after lockdown.

Another longitudinal study [28] conducted in China from October 2019 through March 2020 showed that the hypertensive population in one of the areas hardest hit by the pandemic (Wuhan) experienced a higher increase in SBP during the growth phase of the epidemic in comparison with other less affected areas (with differences between 2.1 and 3 mmHg). However, these parameters again returned to normal or even reached values below pre-pandemic limits within a short period of time.

Spain was one of the European countries hardest hit by the virus. The slight increase in BP parameters observed in our study might be accounted for, in part, by this severity and by the particular susceptibility which persons with AHT, as a risk group, appear to show to the psychological consequences of the pandemic (anxiety, anguish, stress, etc.) [33, 34]. Some previous studies suggest that these stressful situations, the result of home lockdown and isolation, could have increased the activity of the sympathetic nervous system [33–35] and altered the quality of sleep [35, 36], with this in turn having particularly negative consequences on BP. As some works showed [37] the psychological consequences of the pandemic are more likely to be suffer by women, which could explain the greater worsening of blood pressure levels among the female gender in our study.

The nationwide lockdown imposed by the government to halt the propagation of the novel virus, has had an important impact, not only on people’s psychological wellbeing, but also on their lifestyles and daily routines [38, 39]. These restrictive measures have hindered physical activity, thereby increasing exposure to sedentariness, something that could have contributed to the worsening of BP figures [40, 41]. Similarly, the development of unhealthy habits, such as high consumption of alcohol [42] and tobacco [43], during the months of lockdown, might have favored the increase in BP levels. As regards the slight improvements observed in cholesterol and anthropometric measurements, previous studies undertaken in this country during the months of lockdown [44], report healthy dietary changes in the Spanish population (with a higher consumption of fruit and vegetables, and a lower consumption of processed meat and sugar-sweetened beverages).

Control and follow-up of patients with hypertension after the introduction of drug treatment was already inadequate well before the pandemic, according to some studies [45, 46]. The successive waves of the pandemic in Spain have at times overwhelmed the healthcare services, which have destined a great deal of their resources to the diagnosis and treatment of positive cases, and additionally, to the immunization of the population [47]. This state of affairs, taken together with changes in social dynamics resulting from the decision to extend the restrictive measures, may well have delayed the normalization of BP parameters to pre-pandemic levels, as observed in the study by S. Zhang et al [28].

With respect to use of antihypertensive drugs in the 6 months following the end of lockdown, the reduction observed in the total number of DHD dispensed at pharmacies might be partly accounted for by difficulties in access to drug treatments, especially during the first wave [48]. Furthermore, at the outset of the pandemic, some papers suggested that treatment with antihypertensive drugs which act on the renin–angiotensin–aldosterone system might be a risk factor, in terms of severity, for hospitalized patients with COVID-19 infection [49]. Despite the fact that some scientific societies subsequently contradicted this [50], use of these drugs may have been slightly reduced.

Six months after the end of lockdown, PC services continue to receive fewer patients with hypertension than they did in the previous 6 months. Only those patients who received a home visit by their nurse (routine care) experienced an increase in the following 6 months. Fear of becoming infected and a possible worse prognosis, or the collateral effects of lockdown on the health status of older patients with hypertension may have hindered or prevented them from visiting the health center [51].

Similarly, 6 months after the end of strict lockdown, the number of visits to healthcare services (primary and specialized care) and the performance of diagnostic tests on patients with hypertension, have still not reached pre-pandemic levels. These results are consistent with those published by the WHO in its survey of national capacity for the prevention and control of non-transmissible diseases, in which 53% of the countries surveyed reported total or partial interruptions in hypertension-management services as a consequence of the pandemic [52].

With reference to the number of visits to other health center professionals, other studies [53, 54], suggest a decrease in midwife visits during lockdown what it meant a decrease in gynecological cancer diagnosis. This delay might have led to an increase in the number of visit during the following months, which would explain the results of our study. The social and economic impact of the health crisis would account for the increase in visits to PC social services [55]. Lastly, the observed increase in initial visits to specialized care might entail an increase in the number of patients on waiting lists. This could delay the diagnosis of some diseases and favor the development of complications.

As regards the increase in observed mortality in the second 6 months of the study period, the interruption in healthcare services for cardiovascular diseases during the pandemic could have delayed referral, diagnosis, and treatment of these diseases, thus contributing to the excess deaths [56].

### Strengths and Limitations

Our study has some limitations. First, its large sample size means that small variations in these parameters may prove statistically significant.

Second, it is not known whether measurement of BP was exclusively made during nursing visits or whether, in contrast, it was combined with arterial self-measured blood pressure (SMBP) monitoring, something that would allow for a more accurate diagnosis to be made.

Thirdly, we only know the number of patients without a diagnosis of COVID-19, which is different from the number of patients without the disease (real non – infected). Moreover, in the case of some clinical variables, there are very few records. To be able to access these, a GP must validate the data. What this means in the context of this study is that we only had access to measurements which had been validated by GPs.

Furthermore, our study did not include self-reported data on the lifestyles (physical activity, eating habits, tobacco and alcohol consumption, etc.) followed by the population with hypertension during the months of lockdown. Hence, undertaking studies which included qualitative data and information on lifestyles would make it possible to better ascertain the effect of lockdown on the maintenance of daily routines and control of hypertension.

Lastly, it would be of interest to ascertain the trend in the clinical parameters of this disease over a longer period of time (i.e., 12 months after the end of strict lockdown).

### Conclusion

This study contributes, from a longitudinal standpoint, to knowledge of the consequences of lockdown on a sample of patients with AHT but without a diagnosis of COVID-19 infection, by simultaneously considering clinical, pharmacological, and health-resource utilization variables.

The results of our study suggest that the COVID-19 pandemic has hindered access to routine care by patients with chronic diseases such as hypertension. However, the cancellation of medical visits and tests, coupled with changes in lifestyles induced by the pandemic, has not substantially compromised the health and quality of life of these patients.
